# Postpartum depression among women in Nagoya indirectly exposed to the Great East Japan Earthquake

**DOI:** 10.1038/s41598-018-30065-w

**Published:** 2018-08-02

**Authors:** Chika Kubota, Takashi Okada, Mako Morikawa, Yukako Nakamura, Aya Yamauchi, Masahiko Ando, Tomoko Shiino, Masako Ohara, Satomi Murase, Setsuko Goto, Atsuko Kanai, Tomoko Masuda, Branko Aleksic, Norio Ozaki

**Affiliations:** 1 Department of Psychiatry, Nagoya University Graduate School of Medicine, Nagoya, Aichi Japan; 2 Center for Advanced Medicine and Clinical Research, Nagoya University Graduate School of Medicine, Nagoya, Aichi Japan; 3 Liaison Medical Marunouchi, Nagoya, Aichi Japan; 4 Goto Setsuko Ladies Clinic, Nagoya, Aichi Japan; 5 Graduate School of Education and Human Development, Nagoya University, Nagoya, Aichi Japan; 6 Graduate School of Law, Nagoya University, Nagoya, Aichi Japan

## Abstract

This study aimed to assess the situation of postpartum depression and maternal bonding in Nagoya, a city distant from the epicenter of the Great East Japan Earthquake that occurred on March 11, 2011. Among the participants at 1 month after childbirth between March 11, 2010 and March 10, 2013 (n = 188), 152 fully responded to the Edinburgh Postnatal Depression Scale (EPDS) and Mother–Infant Bonding Questionnaire (MIBQ). They were divided into pre-quake (n = 58), and 0–6, 6–12, 12–18, and 18–24 months after the earthquake groups (n = 20, 26, 29, and 19, respectively). The rate of mothers who scored above the cutoff point for the EPDS increased from 12.1% in the pre-quake to 35.0% in the 0–6 months group (p = 0.022). The EPDS total and anxiety subscale scores (mean ± standard error) were also significantly different between the pre-quake and 0–6 months after the earthquake groups (4.45 ± 0.50 vs. 7.95 ± 1.47, p = 0.024; 2.16 ± 0.26 vs. 3.65 ± 0.57, p = 0.021, respectively). The EPDS total and anxiety scores were the highest for the 0–6 months group, followed by the 6–12, 12–18, 18–24 months groups (p = 0.019, p = 0.022). MIBQ scores did not differ between the pre-quake and 0–6 months groups. Depressive symptoms, mainly explained by anxiety, increased after the earthquake with no changes in maternal bonding.

## Introduction

The Great East Japan Earthquake that occurred on March 11, 2011 severely damaged vast areas of Japan. It caused the deaths of approximately 16,000 people, and the subsequent tsunami destroyed as many as 300,000 homes. Such a large-scale disaster increases the risk of mental illness^1^, including postpartum depression (PPD).

A systematic review of 49 papers indicated a significant correlation between the severity of the damage from disasters and PPD^2^. Other than direct destruction by the disaster, psychosocial stress owing to the death of victims, secondary economic damage, witnessing terrifying scenes via media coverage, and so on may be associated with the risk of PPD.

A previous study reported that 21.3% of mothers were suspected of having PPD in Miyagi Prefecture, including those living close to the epicenter of the Great East Japan Earthquake^3^; however, it remains unclear to what degree the Great East Japan Earthquake affected PPD. For example, questions remain regarding the impact of a disaster such as the Great East Japan Earthquake on maternal childcare. PPD is associated with maternal bonding failure^4–6^. Maternal bonding refers to the mother’s loving attitude toward her infant and is an indication of maternal childcare function. Although PPD may be differently related to maternal bonding failure, no previous reports have focused on maternal bonding after a disaster.

Since 2004, we have established a cohort of perinatal women in Nagoya city. Nagoya is about 700 km from the epicenter and was not directly damaged by the Great East Japan Earthquake. However, a massive earthquake (magnitude of 8 or higher) is expected to strike the Tokai area, including Nagoya, with a probability of 70–80% within the next 30 years. After the earthquake, the Fukushima Daiichi nuclear disaster released a large amount of radioactive materials into the environment. Pregnant and postpartum women may more seriously fear the possible exposure of their fetus or newborn children to radiation from contaminated water or food. We hypothesized that perinatal women in this high-risk area might become anxious and depressed more easily because of media coverage or other factors associated with the earthquake. To examine whether such a disaster could affect perinatal women in distant areas from the epicenter, we investigated the relationship between PPD and maternal bonding after the Great East Japan Earthquake.

The strengths of our study are as follows. First, in our previous observational study, we validated the Japanese version of the Edinburgh Postnatal Depression Scale (EPDS)^7^ and revealed its three-factor structure consisting of anxiety, depression, and anhedonia^8^. As a result, the symptomatological features of PPD could be clarified using the EPDS. Second, we also validated the Japanese version of the Mother–Infant Bonding Questionnaire (MIBQ)^9^. Using the MIBQ, we could evaluate the maternal childcare function indicated by maternal bonding after a disaster. Third, we investigated whether the earthquake could affect perinatal women in distant areas from the epicenter.

The aim of this study was to reveal the symptomatological features of PPD and the maternal childcare function indicated by maternal bonding after the disaster in distant areas from the epicenter.

## Methods

### Participants

Our perinatal cohort was established in 2004 at two maternity facilities in Nagoya (Nagoya Teishin Hospital and Kaseki Hospital). Pregnant women attending perinatal class (starting before the 25th week) were given detailed information about the study design and methods. This information was provided both orally and in written form, and informed consent was obtained from each participant. The eligibility criteria were as follows: 20 years of age or older and able to read and write Japanese. Women who agreed to cooperate in the study were asked to complete the EPDS, a self-report questionnaire used for assessing PPD, at 1 month after childbirth and to return it by mail. We also asked participants about their psychosocial backgrounds, including age, years of schooling, income, and parity, at 1 month postpartum.

In the present study, we included those who were at 1 month after childbirth between March 11, 2010 and March 10, 2013 because the Great East Japan Earthquake occurred on March 11, 2011. Among 188 participants who were at 1 month after childbirth during this period, 152 returned completed questionnaires. Participants with more than one childbirth were included only at the first chance to participate in the study. The participants were then divided into five groups based on when they answered the questionnaire: pre-quake (0–12 months before the earthquake), and 0–6, 6–12, 12–18, and 18–24 months after the earthquake.

### EPDS

The EPDS is a 10-item questionnaire developed as a self-report screening tool for PPD^7^. Each question is scored from 0–3, and the total score can range from 0–30. The EPDS has shown good internal consistency (Cronbach’s alpha coefficient = 0.87) and reliability (split-half reliability = 0.88)^7^.

The Japanese version of the EPDS translated by Okano *et al*.^9^ was used in the present study. It was retranslated into English and ascertained to be equivalent to the original. It has also shown good internal consistency (Cronbach’s alpha coefficient = 0.78) and test–retest reliability (Spearman’s correlation = 0.92). Its cutoff point of 8/9 has good sensitivity (75%) and specificity (93%)^9^. Its positive predictive value is 50%. An examination of its factor structure indicated that the three-factor model of anxiety (items 3, 4, and 5), depression (items 7, 8, and 9), and anhedonia (items 1 and 2) was the best-fit model^8^. The cutoff point of 8/9 and the depression, anxiety, and anhedonia subscales were used in the present study.

### MIBQ

The MIBQ was developed by Taylor *et al*. to evaluate maternal bonding between a mother and her baby^10^. It is composed of nine questions, and the total score can range from 0–27. A lower total score indicates better maternal bonding. The MIBQ has shown good internal consistency. Pearson’s correlation coefficients for MIBQ scores can range from 0.77–0.95^11^.

The Japanese version of the MIBQ, which was retranslated into English and ascertained to be equivalent to the original by Yamashita^12^, was used in the present study. The Japanese version of the MIBQ has been validated and found to have good internal consistency (Cronbach’s alpha coefficients: 0.879 and 0.584)^13^.

### Statistical analysis

The participants were divided into the following five groups based on when they answered the questionnaire, either before or sometime after the earthquake: pre-quake group, 0–6 months group, 6–12 months group, 12–18 months group, and 18–24 months group.

Using data from each group, including total EPDS score, scores for each EPDS subscale, and total MIBQ scores, we calculated the mean and standard error (SE) of the scores for each group.

The Kruskal−Wallis test was used to compare age, years of education, and proportion of primiparas between the five groups. The Mann–Whitney U test was used to compare the mean total and subscale (anxiety, depression, and anhedonia) EPDS scores, and the mean MIBQ scores between the pre-quake and 0–6 months groups. The Jonckheere–Terpstra test was used to examine changes over time in the EPDS total and anxiety subscale scores.

### Ethics statement

The study was described to all participants both verbally and in writing, and written informed consent was obtained from each participant. This study protocol was approved by the Ethics Committees of the Nagoya University Graduate School of Medicine, the Ethics Committees of Nagoya Teishin Hospital, and the Ethics Committees of Kaseki Hospital. This study was conducted in accordance with the established ethical standards of all institutions.

### Data availability

The datasets analyzed in the current study are available from the corresponding author on reasonable request.

## Results

Among 188 women who were at 1 month after childbirth between March 11, 2010 and March 10, 2013, 152 returned completed both the EPDS and the MIBQ. The number of participants in each group based on when they answered the questionnaire was as follows; pre-quake (n = 58), 0–6 months (n = 20), 6–12 months (n = 26), 12–18 months (n = 29), and 18–24 months (n = 19).

The average number of days postpartum of the participants was 30.4 (standard deviation [SD]: ± 6.1). Their mean age was 32.1 (SD: ± 6.4) years, and they had a mean of 14.9 (SD: ± 1.5) years of schooling. As for economic status, the most frequent annual income ranged from 4–6 million yen. The mean age, years of schooling, and annual income were almost identical to national averages. Regarding birth history before the most recent birth, 81.3% of the participants were nulliparous, 12.5% were primiparous, 5.3% had second births and 0.7% had third births. None of the women had four or more births. The mean total EPDS score was 5.39 (SE: ± 0.39). The mean EPDS factor subscale scores were 2.56 (SE: ± 0.19) for anxiety, 1.03 (SE: ± 0.12) for depression, and 0.39 (SE: ± 0.87) for anhedonia.

The demographic data for each participant group are shown in Table 1. No significant differences were observed in terms of age, years of education, or proportion of primiparas between the five groups (p = 0.679, 0.297, and 0.933, respectively; Kruskal–Wallis test).

**Table 1:** Demographic data for each participant group.

	Pre-quake	0–6 months	6–12 months	12–18 months	18–24 months	Total	p-values
Participants	n = 58	n = 20	n = 26	n = 29	n = 19	n = 152	—
Age (y) (mean ± SD)	33.0 ± 4.2	31.3 ± 5.2	32.2 ± 4.7	32.7 ± 4.3	29.0 ± 13.5	32.1 ± 6.4	0.679*
Parity: n (%)							
First	47 (81.0)	17 (85.0)	20 (76.9)	25 (85.2)	15 (79.0)	124 (81.3)	0.933*
Second	8 (14.8)	3 (15.0)	4 (15.4)	2 (6.90)	2 (10.5)	19 (12.5)	—
Third	2 (3.4)	0 (0)	2 (7.7)	2 (6.90)	2 (10.5)	8 (5.3)	—
Fourth	1 (1.7)	0 (0)	0 (0)	0 (0)	0 (0)	1 (0.7)	—
Education (y) (mean ± SD)	14.8 ± 1.4	15.1 ± 1.5	14.3 ± 1.8	15.0 ± 1.4	15.2 ± 1.2	14.9 ± 1.5	0.297*
EPDS (mean ± SE)							
Total score	4.45 ± 0.50	7.95 ± 1.47	6.27 ± 1.03	5.38 ± 0.91	4.37 ± 0.77	5.39 ± 0.39	0.024^†^
Anxiety	2.16 ± 0.26	3.65 ± 0.57	2.96 ± 0.50	2.69 ± 0.48	1.89 ± 0.42	2.56 ± 0.19	0.021^†^
Depression	0.79 ± 0.16	1.75 ± 0.48	1.35 ± 0.30	0.83 ± 0.25	0.84 ± 0.18	1.03 ± 0.12	0.059^†^
Anhedonia	0.29 ± 0.73	0.65 ± 1.09	0.27 ± 0.60	0.55 ± 1.18	0.37 ± 0.76	0.39 ± 0.87	0.078^†^
EPDS > 8 (%)	12.1	35.0	34.6	24.1	10.5	21.1	0.022^†^
MIBQ score	1.95 ± 0.29	1.85 ± 0.70	1.73 ± 0.40	1.69 ± 0.36	1.58 ± 0.39	1.80 ± 0.18	0.338^†^

EPDS: Edinburgh Postpartum Depression Scale, MIBQ: Mother-Infant Bonding Questionnaire.

SD: Standard deviation, SE: Standard error.

*p value compared between five groups using the Kruskal–Wallis test.

^†^p value compared between the pre-quake group and the 0–6 months group using the Mann–Whitney U test.

Regarding mothers who scored above the cutoff point for the EPDS and were identified as being at high risk for PPD, there were 12.1% in the pre-quake group, 35.0% in the 0–6 months group, 34.6% in the 6–12 months group, 24.1% in the 12–18 months group, and 10.5% in the 18–24 months group. The proportion of mothers who scored above the cutoff point (8/9) for the EPDS (high risk for PPD) in the 0–6 months group was significantly higher than that in the pre-quake group (p = 0.022; Mann–Whitney U test).

**Figure 1:**
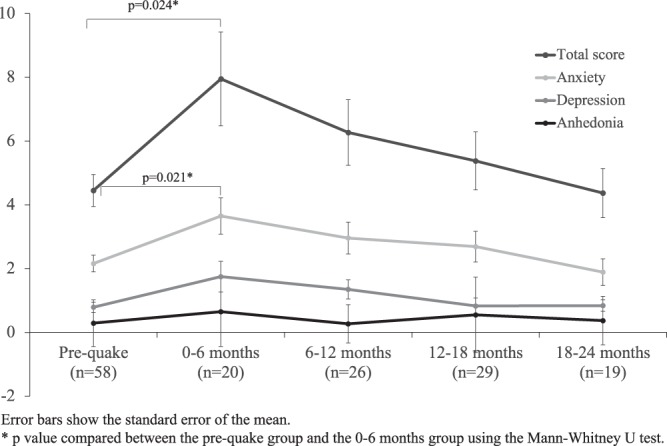
Mean total and subscale scores on the Edinburgh Postnatal Depression Scale.

Figure 1 shows the mean EPDS total and subscale scores in each group. The Mann–Whitney U test was used to compare the EPDS total and subscale (anxiety, depression, and anhedonia) scores between the pre-quake and 0–6 months group; the p values were 0.024, 0.021, 0.059, and 0.078, respectively. The differences in total EPDS and anxiety subscale scores were significant (p = 0.024 and p = 0.021; Mann–Whitney U test).

The total MIBQ score was 1.80 (SE: ± 0.18), and the mean scores of each group were 1.95 (SE: ± 0.29) in the pre-quake group, 1.85 (SE: ± 0.70) in the 0–6 months group, 1.73 (SE: ± 0.40) in the 6–12 months group, 1.69 (SE: ± 0.36) in the 12–18 months group, and 1.58 (SE: ± 0.39) in the 18–24 months group. The total MIBQ score did not differ between the pre-quake and 0–6 months groups (p = 0.338; Mann–Whitney U test).

Based on the results of the Jonckheere–Terpstra test, the 0–6 months group had the highest total EPDS and anxiety scores, followed by the 6–12 months group, the 12–18 months group, and the 18–24 months group (p = 0.019 and 0.022, respectively).

## Discussion

To our knowledge, this is the first observational, cross-sectional study to examine the symptomatological features of PPD and maternal bonding in areas indirectly exposed to the Great East Japan Earthquake.

The four main findings of the present can be summarized as follows: 1) The rate of mothers suspected of having PPD increased after the earthquake; 2) The EPDS total scores increased sharply in participants within 6 months after the earthquake, but then decreased over the subsequent 18 months; 3) The anxiety subscale scores increased after the earthquake, but then decreased over the subsequent 18 months (the differences in scores for depression and anhedonia before and after the earthquake were not significant); and 4) The MIBQ scores before and after the earthquake were not significantly different.

As expected, the rate of women suspected of having PPD increased after the Great East Japan Earthquake. Similar results were found in a study of PPD in Miyagi Prefecture, including the epicenter, after the Great East Japan Earthquake^3^. However, that study did not examine the EPDS subscales. It should be noted that in the present study, only symptoms of anxiety increased the rate of PPD. Consequently, it may be necessary to distinguish disaster-related PPD from general PPD.

The increase in anxiety scores after the earthquake was as expected. Some studies have shown a relationship between depression and anxiety symptoms during the postpartum period after a disaster. Victims of serious flooding in the United States were reported to have anxiety symptoms^14^, and a cohort study revealed that women who had severe symptoms of anxiety were more vulnerable to PPD (odds ratio = 3.75) after an earthquake in Armenia^15^. Similar to these previous studies, our findings show that mothers affected by the Great East Japan Earthquake exhibited anxiety symptoms.

However, our results showed that the severity of anxiety decreases to pre-earthquake levels after 2 years. As no particular psychotherapeutic interventions were required to improve the anxiety symptoms, and no changes were seen in the depression and anhedonia symptoms, depressive symptoms after the earthquake could be explained by anxiety as a reaction to a disaster.

Unexpectedly, our findings showed that maternal bonding did not change after the earthquake. According to our previous study, PPD significantly affects bonding between a mother and child in non-disaster situations^4^. It is possible that stronger maternal bonding is a natural reaction, as mothers attempt to protect their children during unexpected situations. Some studies have pointed out that maternal bonding improves during some stressful situations. For example, in a systematic review, five of 18 studies showed an equal or higher quality of mother–infant bonding in preterm groups compared with full-term groups^16^. Another study showed that mothers with pregnancy complications exhibited better bonding^17^. The Great East Japan Earthquake may be a similar stressful situation in terms of enhancing maternal bonding.

As PPD after the earthquake did not affect maternal bonding in the present study, the anxiety experienced after the disaster could have been a normal reaction to an unexpected situation and therefore may not have required psychotherapeutic intervention. The need of mothers for mental health care to improve the mother–infant relationship is considered low. However, the cause-and-effect relationship between maternal bonding and PPD remains unclear.

Although the compared groups did not differ in terms of several sociodemographic characteristics, possible major determinants of PPD were not adjusted for in this study. A meta-analysis revealed that the predictors of PPD include prenatal depression, self-esteem, childcare stress, prenatal anxiety, life stress, social support, marital relationship, history of previous depression, infant temperament, maternity blues, marital status, socioeconomic status, and unplanned/unwanted pregnancy^18^. Some of these sociodemographic characteristics may have changed after the disaster, even in indirectly affected areas. In addition, neonate characteristics (gender, gender satisfaction, child number, infant weight, health problems in infant) may affect PPD^19^. In the present study, we did not collect data on psychosocial status, history of mental illness, physical health conditions, family history of mental illness, or health of the neonate. For a more comprehensive examination of PPD after a disaster, psychiatric statuses other than a depressive state and other possible confounding factors in regard to psychosocial backgrounds should be considered in future studies.

The present study had some additional limitations. First, the sample size of the groups after the earthquake was small, which increases the possibility of a type II error. Second, we did not measure what kind of earthquake-related adversities were experienced by the perinatal women. Therefore, the adversities affecting perinatal women remain unclear, as is the relationship between EPDS scores and specific adversities related to the earthquake. Third, some confounding factors may be possible, which makes it difficult to conclude that the observed tendencies were solely the result of the devastating earthquake. Fourth, there was a possibility of selection bias. The participants in this study were recruited from two standard medical facilities in the central area of Nagoya. This geographical characteristic may have had some influence on the results. However, the participants’ demographics did not differ much from national averages. Fifth, we did not obtain any PPD data after the earthquake in other distant areas from the epicenter. Therefore, we cannot conclude whether our findings are specific to Nagoya or common to other distant areas. Lastly, we cannot compare our results with the data sampled in the directly affected area because the data collection time points differed.

In conclusion, an increase in the rate of PPD was observed in areas indirectly exposed to the Great East Japan Earthquake. This was thought to be the result of anxiety as a reaction to the disaster and was not associated with any changes in maternal bonding.
